# Androgen-Receptor Positive Lacrimal Sac Adenocarcinoma Demonstrating Long-Lasting Response to LHRH Analog Plus Abiraterone Treatment

**DOI:** 10.3389/fonc.2015.00010

**Published:** 2015-02-02

**Authors:** Elena Vagia, Panagiota Economopoulou, Nikolaos Oikonomopoulos, Ilias Athanasiadis, George Dimitriadis, Amanda Psyrri

**Affiliations:** ^1^Oncology Unit, 2nd Department of Internal Medicine – Propaedeutic, Attikon University Hospital, Haidari, Greece; ^2^2nd Department of Radiology, Attikon University Hospital, Haidari, Greece; ^3^Department of Oncology, Hygeia Hospital, Athens, Greece

**Keywords:** lacrimal sac, adenocarcinoma, androgen receptor, abiraterone, molecular target

## Abstract

Tumors arising at the lacrimal sac are extremely rare, as a limited number of cases have been reported worldwide. They are commonly primary and the majority of them are malignant and epithelial in origin. Adenocarcinomas account for a small percentage of these tumors. Treatment of local disease mainly includes complete surgical resection. However, metastatic disease has a poor prognosis and the development of new treatment strategies is highly important. Research efforts mainly focus on the identification of molecular targets for therapy. Herein, we describe for the first time a case of a patient with an androgen receptor expressing adenocarcinoma of the lacrimal sac that had an impressive response to abiraterone.

## Background

The tumors of the lacrimal drainage system are extremely rare as approximately 400 cases have been reported in the literature to date (1–3). The lacrimal sac is the upper dilated end of the nasolacrimal duct formed by the lacrimal bone and the frontal process of the maxilla (2).

Tumors arising at the lacrimal sac are usually primary. Most of these tumors are malignant (75%) and of epithelial origin (70%) including squamous cell carcinoma, transitional cell carcinoma, mucoepidermoid, adenoid cystic, poorly differentiated carcinoma, and adenocarcinoma. The non-epithelial histologies include lymphoma, histiocytoma, sarcoma, hemangiopericytoma, malignant melanoma, and neurofibroma (4). Secondary tumors usually arise from adjacent organs (orbit, paranasal sinuses). Mortality rate depends on histology and stage. Early detection and treatment are very important for this category of tumors (5).

Lacrimal sac tumors usually present with symptoms such as epiphore, dacryocystitis, and pain with or without associated tumor mass. Differential diagnosis includes infection or inflammatory lesions (dacryocystitis, tuberculosis, fungal infections, Wegener’s granuloma, or sarcoidosis). CT and MRI of the head and neck are required for the evaluation of the size of the tumor mass, the extent of the invasion into the adjacent structures and assessment of regional lymph node status. Chest imaging is also required at diagnosis as these tumors often metastasize to the lungs (6).

With the exception of lymphomas, lacrimal sac carcinomas should be treated with complete surgical removal of the tumor with wide excision to achieve clear margins. Resection of adjacent structures and lymph node dissection may be required in more extensive disease (2). For epithelial tumors, post-operative radiotherapy is usually recommended (5). Local recurrence may be treated with surgery, radiotherapy, or chemotherapy. Metastatic disease is associated with a very poor prognosis. In the era of molecular targeted therapies, new therapeutic approaches are under evaluation (2, 3, 5). In this report, we present a case of impressive response of an androgen receptor (AR) expressing lacrimal sac adenocarcinoma to abiraterone.

## Case Presentation

In May 2007, a 65-year-old man was diagnosed with locally advanced lacrimal cyst adenocarcinoma. The patient presented with dacryorrhea and 3 months later, he developed a right parotid mass and multiple enlarged cervical lymph nodes. He underwent a complete surgical resection including right ophthalmus, right parotid gland, and lymph node dissection. Biopsy revealed a lacrimal sac adenocarcinoma with positive surgical margins. Post-operative radiotherapy was administered (50 Gy) with concomitant administration of weekly cisplatin at a dose of 35 mg/m^2^. Baseline CEA level before surgical resection was not evaluated. After surgical resection, CEA level was 13 mg/L.

Two years later, he developed recurrence manifested with extensive superficial cutaneous involvement of the right cervical area. Biopsy confirmed recurrence of a lacrimal sac adenocarcinoma. The tumor cells were positive for EGFR (3+), AR (strongly positive) HER-2 expression (2+) by immunohistochemistry. FISH was positive for HER-2 amplification. Six cycles of carboplatin/docetaxel/trastuzumab were administered every 3 weeks between May and September of 2009. The patient attained a partial response (PR), which was short-lived. Two months following completion of chemotherapy, the patient had disease progression with appearance of new cutaneous lesions. Second line chemotherapy with eight cycles of cisplatin/pemetrexed/bevacizumab every 3 weeks was administered between November 2009 and April 2010. A complete clinical and radiological remission was achieved and maintenance therapy with bevacizumab was given until disease progression.

Six months after completion of cisplatin/pemetrexed and bevacizumab triplet and while on bevacizumab maintenance, the patient developed new cutaneous nodules at the right neck area, which were positive for adenocarcinoma on fine needle aspiration. Third line chemotherapy with weekly carboplatin/paclitaxel/cetuximab was initiated. Following the administration of a single dose, the patient developed grade III mucosal toxicity and grade IV diarrhea that led to omission of cetuximab in subsequent treatment cycles. After four cycles of chemotherapy, the patient was deemed to have PR and a decision was made for him to discontinue chemotherapy and proceed to electron beam radiotherapy, which was administered between February and March 2011.

In April 2012, a PET–CT identified osteoblastic bone metastasis. Administration of afatinib and zolendronic acid was begun. After 4 months of treatment, afatinib was discontinued due to disease progression, which involved presence of new cutaneous nodules in the right zygomatic area and soft tissue mass in the temporal area. In January 2013, the patient presented to our hospital for the first time for a second opinion. Due to AR positivity, treatment with bicalutamide and LHRH was begun. The patient displayed PR to androgen ablation, which was manifested as size reduction of the temporal mass and decline in CEA level (from 13 to 10 mg/L), which was unfortunately short-lived, as it lasted for only 2 months.

**Figure 1:**
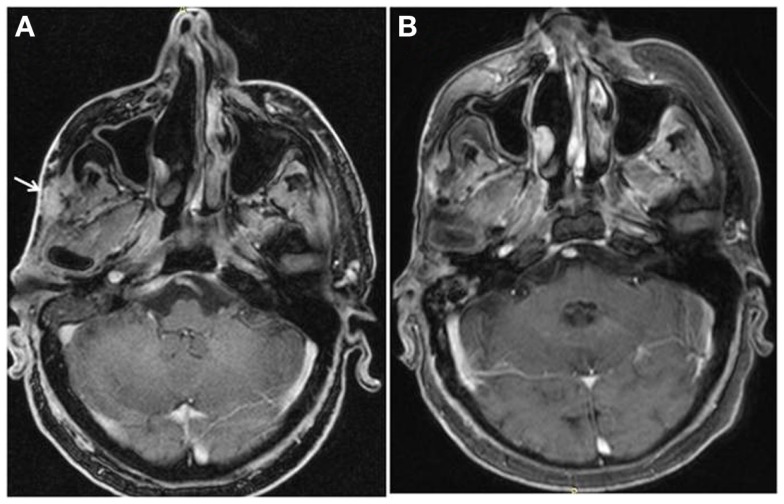
**Complete resolution of temporal soft tissue mas after six months of treatment with abiraterone**.

In August 2013, bicalutamide was substituted by abiraterone (1000 mg daily) plus prednisolone (10 mg daily), while LHRH analog was continued. Disease evaluation at 3 months revealed a good clinical response with disappearance of cutaneous lesions and a decrease in size of the temporal soft tissue mass, as well as a substantial biochemical response (CEA level dropped from 10 to 4 mg/L). After 6 months of treatment, the patient had a complete response according to RECIST criteria, with resolution of cutaneous lesions and temporal soft tissue mass (Figure 1). At the last follow-up in August 2014, clinical, biochemical, and imaging evaluation showed no evidence of disease progression and the patient was continued on abiraterone/LHRH treatment.

## Discussion

Herein, we describe for the first time a case of adenocarcinoma of the lacrimal sac treated successfully with abiraterone. Abiraterone inhibits 17 α-hydroxylase/C17,20 lyase (CYP17A1), an enzyme mainly expressed in testicular, adrenal, and prostatic tumor tissues. More specifically, CYP17 catalyzes two sequential reactions; the conversion of pregnenolone and progesterone to their 17-α-hydroxy derivatives by its hydroxylase activity, and the subsequent formation of dehydroepiandrosterone (DHEA) and androstenedione, respectively, by its lyase activity. DHEA and androstenedione are androgens and precursors of testosterone. As a result, inhibition of CYP17 activity by abiraterone decreases circulating levels of testosterone (7). Abiraterone is approved by both Food and Drug Administration (FDA) and European Medicines Agency (EMA) in combination with prednisone for treatment of patients with metastatic castration-resistant prostate cancer.

Tumors of the lacrimal sac are very rare and heterogeneous. Surgical resection is the backbone of treatment when a total resection of the tumor is feasible. Radiotherapy and chemotherapy may be added. When a tumor recurs locally or distantly and surgery or radiotherapy is not therapeutic options, systemic therapy can be considered. However, administration of chemotherapy is anecdotal and has been disappointing (2).

Adenocarcinomas constitute a small percentage of tumors of the lacrimal sac. These are rare tumors and research efforts focus on identification of molecular targets for therapy. In our case, the tumor was found to be AR positive. This is in concordance with one previous report (8). Other molecular targets for therapy may include HER-2 and mTOR (8, 9).

The lacrimal and salivary glands share similar characteristics. Lacrimal sac adenocarcinomas may originate from seromucinous glands in the lacrimal sac. These glands constitute of serous and mucinous units and have been found to be similar in salivary gland and lacrimal sac (10). Furthermore, salivary gland type neoplasms, such as adenoid cystic carcinoma, mucoepidermoid carcinoma, and pleomorphic adenoma, have been reported in the lacrimal sac and these tumors might arise from the seromucinous glands in the lacrimal sac (2–4). Therefore, salivary gland and lacrimal sac tumors might be of the same origin.

Androgen-deprivation therapy consisting of bicalutamide and LHRH analog has been repeatedly reported to be an effective treatment option for AR+ adenocarcinomas of the salivary glands (11–13). In 2003, Locati et al. first described a patient with AR expressing salivary gland adenocarcinoma who experienced a complete remission 2 months after receiving complete anti-androgen blockade with LHRH analog and bicalutamide (11). More recently, Soper et al. reported a case of a patient with locally advanced AR positive salivary duct carcinoma who had a long-term complete remission shortly after administration of androgen-deprivation therapy (12). Similarly, a case of a patient with a metastatic AR-positive salivary duct carcinoma who had an impressive response with bicalutamide has been reported (13). However, the experience with abiraterone is limited in this setting. In a recent report by Locati et al., abiraterone showed remarkable activity in two patients with AR expressing recurrent and metastatic salivary gland carcinoma (14). In our case, complete anti-androgen blockade with bicalutamide plus LHRH was chosen as the optimal therapy at the time, based on AR expression of the tumor and reported literature on salivary gland carcinomas. Treatment resulted in partial, albeit short-lived response. However, the patient demonstrated an impressive long-lasting response to abiraterone plus prednisolone, which is still ongoing. Interestingly, response to abiraterone was strikingly better to previously administrated chemotherapy, although the patient was heavily pretreated. The choice of abiraterone as appropriate treatment was based on AR signaling and the model of prostate cancer. The genetic background of the tumor underlying this response including the dissection of the molecular events of the androgen signaling pathway is worth exploring.

Of note, potential side effects of abiraterone are concerning, since grade 3 or 4 adverse events have been reported in almost 25% of patients in large randomized trials (15). Coadministration with the recommended dose of glucocorticoid prednisone compensates for abiraterone-induced reductions in serum cortisol and blocks the compensatory increase in adrenocorticotropic hormone seen with abiraterone (16). In general, glucocorticoid-related adverse events tend to occur at doses and/or treatment durations greater than the low dose of glucocorticoid approved in combination with abiraterone acetate.

In the era of molecular targeted therapy, we recommend for all recurrent/metastatic epithelial lacrimal sac tumors to be tested for AR positivity. Potential administration of androgen-deprivation therapy or abiraterone to female patients with AR expressing tumors is intriguing. A randomized study for assessment of effectiveness of abiraterone in lacrimal sac tumors is warranted, albeit extremely difficult to realize due to rarity of the tumor.

## Concluding Remarks

In conclusion, androgen-deprivation therapy including the new anti-androgens such as abiraterone and enzalutamide may prove effective treatment options for AR+ lacrimal gland tumors.

## Author Contributions

Elena Vagia reviewed the literature and wrote the paper. Panagiota Economopoulou and Ilias Athanasiadis collected the data. Nikolaos Oikonomopoulos prepared the figure. Amanda Psyrri and George Dimitriadis carried out critical interpretations and contributed in the final version of the paper. All authors read and approved the final manuscript.

## Conflict of Interest Statement

The authors declare that the research was conducted in the absence of any commercial or financial relationships that could be construed as a potential conflict of interest.

## References

[1] Brannan PA Kersten RC Schneider S Kulwin DR. A case of primary adenocarcinoma of the lacrimal sac. Orbit (2005) 24:291–3. 10.1080/01676830500182739 16354642

[2] Montalban A Lietin B Louvrier C Russier M Kemeny JL Mom T Malignant lacrimal sac tumors. Eur Ann Otorhinolaryngol Head Neck Dis (2010) 127:165–72. 10.1016/j.anorl.2010.09.001 21036121

[3] Stefanyszyn MA Hidayat AA Pe’er JJ Flanagan JC. Lacrimal sac tumors. Ophthal Plast Reconstr Surg (1994) 10:169–84. 10.1097/00002341-199409000-00005 7947444

[4] Pe’er JJ Stefanyszyn M Hidayat AA. Nonepithelial tumors of the lacrimal sac. Am J Ophthalmol (1994) 118:650–8. 10.1016/S0002-9394(14)76580-8 7977578

[5] Parmar DN Rose GE Management of lacrimal sac tumours. Eye (Lond) (2003) 17:599–606 10.1038/sj.eye.6700516 12855966

[6] Ni C D’Amico DJ Fan CQ Kuo PK Tumors of the lacrimal sac: a clinicopathological analysis of 82 cases. Int Ophthalmol Clin (1982) 22:121–40 10.1097/00004397-198202210-00010 6277817

[7] Attard G Belldegrun AS de Bono JS Selective blockade of androgenic steroid synthesis by novel lyase inhibitors as a therapeutic strategy for treating metastatic prostate cancer. BJU Int (2005) 96:1241–6 10.1111/j.1464-410X.2005.05821.x 16287438

[8] Ishida M Iwai M Yoshida K Kagotani A Kohzaki H Arikata M Primary ductal adenocarcinoma of the lacrimal sac: the first reported case. Int J Clin Exp Pathol (2013) 6(9):1929–34. 24040460 PMC3759502

[9] Skalova A Starek Kucerova V Szepe P Plank L. Salivary duct carcinoma – a highly aggressive salivary gland tumor with HER-2/neu oncoprotein overexpression. Pathol Res Pract (2001) 197:621–6. 10.1078/0344-0338-00136 11569926

[10] Pe’er J Hidayat AA Ilsar M Landau L Stefanyszyn MA. Glandular tumors of the lacrimal sac. Their histopathologic patterns and possible origins. Ophthalmology (1996) 103:1601–5. 10.1016/S0161-6420(96)30457-0 8874432

[11] Locati LD Quattrone P Bossi P Marchiano AV Cantu G Licitra L A complete remission with androgen-deprivation therapy in a recurrent androgen receptor-expressing adenocarcinoma of the parotid gland. Ann Oncol (2003) 14:1327–8 10.1093/annonc/mdg331 12881399

[12] Soper MS Iganej S Thompson LD. Definitive treatment of androgen receptor-positive salivary duct carcinoma with androgen deprivation therapy and external beam radiotherapy. Head Neck (2014) 36:E4–7. 10.1002/hed.23383 23720164

[13] Yamamoto N Minami S Fujii M. Clinicopathologic study of salivary duct carcinoma and the efficacy of androgen deprivation therapy. Am J Otolaryngol (2014) 35(6):731–5. 10.1016/j.amjoto.2014.07.007 25087467

[14] Locati LD Perrone F Cortelazzi B Imbimbo M Bossi P Potepan P Activity of abiraterone in rechallenging two AR-expressing salivary gland adenocarcinomas, resistant to androgen-deprivation therapy. Cancer Biol Ther (2014) 15:678–82. 10.4161/cbt.28410 24618694 PMC4049783

[15] Sternberg CN Castellano D Daugaard G Geczi L Hotte SJ Mainwaring PN Abiraterone acetate for patients with metastatic castration-resistant prostate cancer progressing after chemotherapy: final analysis of a multicentre, open-label, early-access protocol trial. Lancet Oncol (2014) 15:1263–8. 10.1016/S1470-2045(14)70417-6 25242048

[16] Yang LP. Abiraterone acetate: in metastatic castration-resistant prostate cancer. Drugs (2011) 71:2067–77. 10.2165/11208080-000000000-00000 21985170

